# New findings on retinal microvascular changes in patients with primary COVID-19 infection: a longitudinal study

**DOI:** 10.3389/fimmu.2024.1404785

**Published:** 2024-05-21

**Authors:** Chenxi Zhang, Shiyu Cheng, Huan Chen, Jingyuan Yang, Youxin Chen

**Affiliations:** ^1^ Department of Ophthalmology, Peking Union Medical College Hospital, Chinese Academy of Medical Sciences & Peking Union Medical College, Beijing, China; ^2^ Key Laboratory of Ocular Fundus Diseases, Chinese Academy of Medical Sciences & Peking Union Medical College, Beijing, China

**Keywords:** coronavirus disease 2019, retinal microvasculature, vessel density, foveal avascular zone, optical coherence tomography angiography

## Abstract

**Purpose:**

To investigate the longitudinal alterations of retinal microvasculature in patients with primary coronavirus disease 2019 (COVID-19) infection.

**Methods:**

A cohort of participants, who had never been infected with COVID-19, was recruited between December 2022 and May 2023 at Peking Union Medical College Hospital in Beijing, China. Participants underwent comprehensive ophthalmologic examinations and fundus imaging, which included color fundus photography, autofluorescence photography, swept-source optical coherence tomography (SS-OCT) and SS-OCT angiography (SS-OCTA). If participants were infected with COVID-19 during the study, follow-ups with consistent imaging modality were conducted within one week and two months after recovery from the infection.

**Results:**

31 patients (61 eyes), with a mean age of 31.0 ± 7.2 years old, were eligible for this study. All participants contracted mild COVID-19 infection within one month of baseline data collection. The average period was 10.9 ± 2.0 days post-infection for the first follow-up and 61.0 ± 3.5 days for the second follow-up. No clinical retinal microvasculopathy features were observed during the follow-ups. However, SS-OCTA analysis showed a significant increase in macular vessel density (MVD) from 60.76 ± 2.88% at baseline to 61.59 ± 3.72%(p=0.015) at the first follow-up, which subsequently returned to the baseline level of 60.23 ± 3.33% (p=0.162) at the two-month follow-up. The foveal avascular zone (FAZ) remained stable during the follow-ups with areas of 0.339 ± 0.097mm^2^, 0.342 ± 0.093mm^2^, and 0.344 ± 0.098mm^2^ at the baseline, first follow-up (p=0.09) and second follow-up (p=0.052), respectively. Central macular thickness, cube volume and ganglion cell-inner plexiform layer showed a transient decrease at the first follow-up(p<0.001, p=0.039, p=0.002, respectively), and increased to baseline level at the two-month follow-up(p=0.401, p=0.368, p=0.438, respectively).

**Conclusion:**

Mild COVID-19 infection may temporarily and reversibly impact retinal microvasculature, characterized by a transient increase in retinal blood flow during the early recovery phase, which returns to the pre-infection level two months post-infection.

## 1 Introduction

The coronavirus disease 19 (COVID-19) pandemic, caused by severe acute respiratory syndrome coronavirus 2 (SARS-CoV-2) has led to about 768 million confirmed infections and approximately 7 million deaths globally according to the World Health Organization since 2020 (1). Although the WHO declared that COVID-19 was no longer a public health emergency of international concern on 5 May 2023, it is still an ongoing global health issue since SARS-CoV-2 will continue circulating widely and evolving (2).

SARS-CoV-2, the virus responsible for COVID-19, has been associated with multi-organ damages, including the eye (3, 4). Retinal microvascular abnormalities, such as hemorrhages, cotton wool spots(CWS), tortuous vessels, and retinal vascular diseases, including retinal vein occlusion (5–7) and paracentral acute middle maculopathy (8) have been reported in COVID-19 patients, with potential implications for severe visual impairment (9, 10). This is postulated to occur due to hypercoagulability and inflammation associated with COVID-19 and that the retina's high demand for oxygen makes it particularly susceptible to microvascular thrombosis (11).

Optical coherence tomography angiography (OCTA), a non-invasive technique, allows for quantification of retinal microvasculature and assessment of disease impact on retinal perfusion. OCTA findings in COVID-19 patients were inconsistently reported in previous studies, most of which were case-control studies. Some studies demonstrated retinal perfusion deficits, such as decreased vessel density (VD) and increased foveal avascular zone (FAZ) area in COVID-19 patients (12–14), whereas some studies showed that there were no such changes (15, 16). The inconsistency could be attributed to factors such as disease severity, age, comorbidities, and self-reported infection status. The imbalance of these factors between patients and controls may induce bias for case-control studies (17). Ideally, prospective cohort studies with laboratory-validated infection status would be implemented, but such studies were previously considered challenging to implement during the COVID-19 pandemic (17).

However, with China's reopening in December 2022 after having adhered to dynamic zero-COVID-19 policy for nearly 3 years (18), we acquired an opportunity to conduct a cohort study on COVID-19-related retinal microvasculopathy in the Chinese population. This research aims to illustrate COVID-19-related retinal microvasculature changes by comparing fundus imaging pre and post-primary infection, follow the progression pattern, and reveal potential underlying pathophysiological mechanisms of this condition.

## 2 Method

### 2.1 Participants

This observational cohort study was conducted over six months at Peking Union Medical College Hospital, Beijing, China, a tertiary academic hospital. Physicians and nurses who had not previously been infected with COVID-19 were invited to participate. This project was approved by the Institutional Review Board of Peking Union Medical College Hospital (study code K4168) and was conducted following the Declaration of Helsinki. Written informed consent was obtained from all participants.

The inclusion criteria were as follows: 1) age ≥18 years; 2) without previous COVID-19 infection history and negative results of real-time, reverse transcription polymerase chain reaction(PCR) of nasopharyngeal swab samples on the day of baseline collection; and 3) voluntary to participate and undergo ophthalmologic examination, color fundus photography, autofluorescence photography, swept source-optical coherence tomography (SS-OCT) and swept source-optical coherence tomography angiography (SS-OCTA) for this research investigation.

The exclusion criteria were as follows: 1) patients with a history of macular disease; 2) patients who had undergone intraocular surgery or retinal laser treatment within six months of evaluation; 3) poor quality of color fundus photography, autofluorescence images, or SS-OCT/SS-OCTA images (quality index <7); 4) current pregnancy or breastfeeding; 5) subjects with serious systemic diseases (tumor, stroke, dementia, etc.).

### 2.2 Study protocol

At baseline, demographic data including gender, age, type and timing of COVID-19 vaccination, and ocular and systemic history were recorded for all participants. Standardized ophthalmic examinations including best-corrected visual acuity (BCVA), intraocular pressure, and slit-lamp microscopy were performed in both eyes.

For fundus imaging examination, all patients underwent color fundus photography (Carl Zeiss Meditec AG, Jena, Germany), autofluorescence photography (Heidelberg Engineering Inc, Heidelberg, Germany), SS-OCT/SS-OCTA (VG200, SVision Imaging, Ltd., Luoyang, China). The SS-OCT instrument operates a scanning laser with a central wavelength of 1050 nm and an A-scan rate of 200,000 scans per second, and 6*6mm macular raster scan was employed in the study. The imaging examinations were performed by the same experienced ophthalmic technician.

If participants got infected with COVID-19 within six months after the baseline data collection (verified with positive SARS-CoV-2 PCR or antigen testing results), follow-ups were conducted within one-week and at two-month after recovery from the infection (defined as twice negative PCR or antigen test results with an interval of at least 24 hours). Ophthalmic and fundus imaging examinations were reperformed at each post-infection visit using the same modalities as baseline. Follow-up mode was used in SS-OCT/SS-OCTA to ensure consistent scan location between pre-and post-scan examinations. However, if the participants remained free of COVID-19 infection during the six-month follow-up period, the examinations were repeated at the end of follow-up (**Supplementary Figure S1**).

### 2.3 Image analysis

Fundus photographs, autofluorescence images, SS-OCT, and SS-OCTA scans were independently reviewed by CXZ and SYC. Poor quality images due to media opacities or acquisition artifacts and SS-OCT/SS-OCTA scans with a signal strength under 7 were excluded. Each participant could contribute up to two eyes to the analysis.

Fundus photography and autofluorescence images were reviewed for the presence of retinal microvasculopathy signs (retinal hemorrhages, CWS and vascular tortuosity) and autofluorescence abnormalities. SS-OCT/SS-OCTA images were automatically segmented by VG200D’s van Gogh software (SVision Imaging, Ltd., Luoyang, China). Segmentation errors were checked and corrected manually before interpreting the images.

SS-OCTA findings included FAZ area and macular vascular density(MVD) of inner retina, which were the main outcome measures. MVD at the level of superficial capillary plexus(SCP), intermediate capillary plexus (ICP) and deep capillary plexus (DCP) were measured as well respectively. The MVD was automatically calculated according to the Early Treatment of Diabetic Retinopathy Study (ETDRS) grid and exported using VG200D’s van Gogh software (SVision Imaging, Ltd., Luoyang, China). The macular zone was divided into regions consisting of three concentric rings with diameters of 1 mm (central fovea), 3 mm (inner ring), and 6mm(outer ring). In the software, MVD corresponded to the percentage of the area occupied by vessels and capillaries based on adaptive thresholding binarization within the analyzed region.

Structural OCT parameters including central macular thickness(CMT), cube volume(CV) and macular average ganglion cell-inner plexiform layer(GCIPL) thickness were also automatically measured according to ETDRS grid and exported using the same software for OCTA images.

### 2.4 Statistical analysis

Statistical analysis was performed using the software SPSS 25.0 (IBM, Chicago, IL, USA). The means and standard deviations of all continuous variables are presented. The normality of data was examined by the Shapiro–Wilk test. The categorical data were evaluated with the chi-square test or Fisher’s exact test. Paired t-tests or Wilcoxon signed-rank tests were used to assess changes in retinal structural and blood flow measurements at baseline and after COVID-19 infection in the same eye. *p* < 0.05 was considered statistically significant.

## 3 Results

32 participants (64 eyes) enrolled in December 2022 were reviewed as eligible for this study. After excluding one participant (two eyes) for missing follow-up and one eye for inadequate SS-OCTA image quality, 61 eyes from 31 participants remained for analysis. The cohort consisted predominantly of healthy individuals, with only one case of hypertension and hyperlipidemia, both well managed with medication. The mean(SD) age of participants was 31.0(7.2) years (range 23–59), with males comprising 41.9% (13/31) of the cohort. 93.5% (29/31) of participants had a history of COVID-19 vaccination (inactive vaccine, either Beijing Institute of Biological Research or Sinovac Life Science Ltd). At baseline, all participants had good ocular condition with BCVA of 20/20 or above and normal intraocular pressure.

All participants were successively infected with SARS-COV-2 within one month of baseline data collection, as confirmed by PCR or rapid antigen tests. All participants experienced fever (≥37.3°C) with a mean(SD) duration of 3.1(1.3) days, among whom 13 patients (41.9%) developed high fever(≥39.1°C). Cough (93.5%), pharyngalgia (87.1%), and nasal congestion (83.9%) were the most common systemic symptoms. None required hospitalization or steroid treatment for COVID-19.

Follow-ups were performed within one week and at two months after recovery from the infection. Calculated by tracing back to the initial day when the infection was confirmed, the first follow-up took place on average 10.9 ± 2.0 days after the day of infection, and the second follow-up was performed on average 61.0 ± 3.5 days post-infection. No significant changes were found in visual acuity and intraocular pressure between follow-up visits and the baseline. No retinal abnormalities were observed in fundus photography and autofluorescence images in follow-up visits. The details are shown in **Table 1**.

**Table 1:** Demographic and Clinical Features of study population(n=31).

Features	No (%)
Sex	
Male	13 (41.9)
Female	18 (58.1)
**Age, years (Mean [SD])**	31.0[7.2]
Covid-19 vaccination history	
Not vaccinated	2 (6.5)
One dose	0 (0)
Two doses	14 (45.2)
Three doses	15 (48.4)
Comorbidities	
None	29 (93.5)
Hypertension	1 (3.2)
Hyperlipidemia	1 (3.2)
**Participants infected with COVID-19**	31 (100)
First follow-up period, days(Mean[SD])*	10.9[2.0]
Second follow-up period, days(Mean[SD])*	61.0[3.5]
Symptoms during infection	
Fever	31 (100)
Duration of fever, days(mean[SD])	3.1[1.3]
High Fever(≥39.1°C)	13 (41.9)
Cough	29 (93.5)
Pharyngalgia	27 (87.1)
Nasal congestion	26 (83.9)
Hypodynamia	25 (80.6)
Headache	24 (77.4)
Muscle soreness	24 (77.4)
Arthrodynia	12 (38.7)
Nausea or vomiting	10 (32.3)
Hypogeusia	5 (16.1)
Abdominal pain or diarrhea	2 (6.5)

* The follow-up period begins on the day of COVID-19 infection.

**Figure 1:**
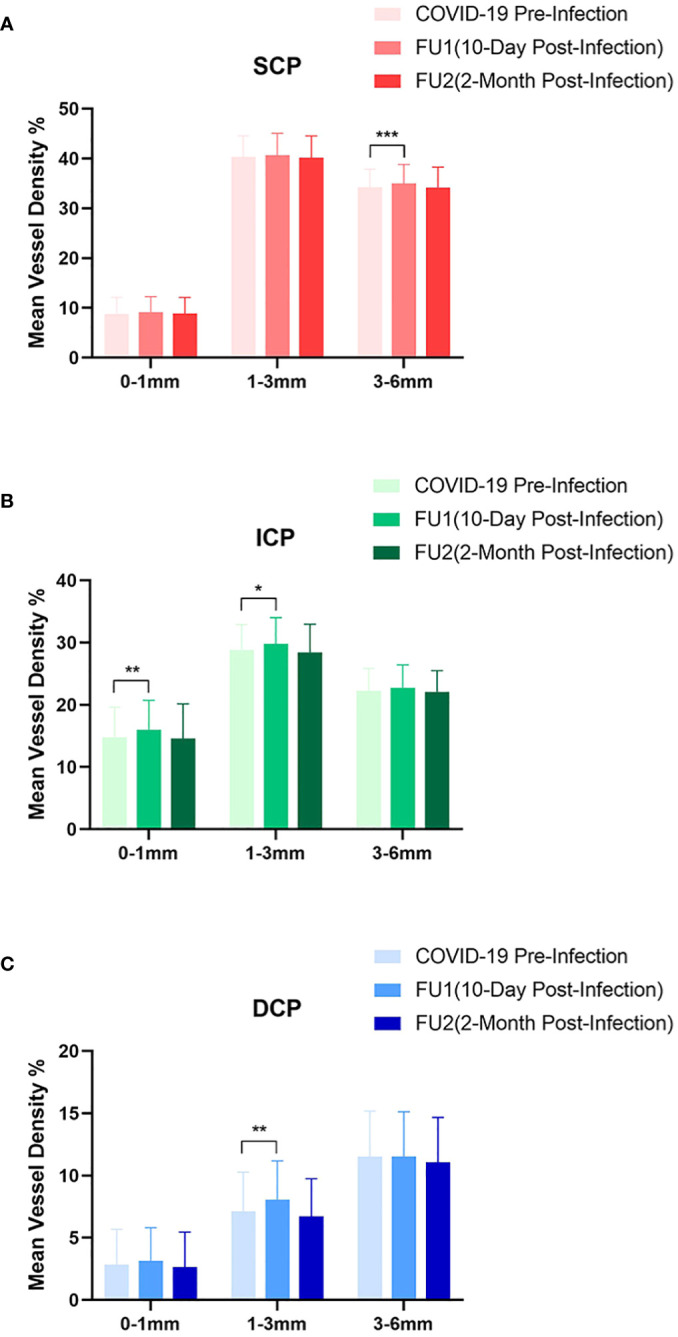
Analysis of macular vessel density on swept-source optical coherence tomography angiography. The macular region were further divided into **(A)** superficial capillary plexus(SCP), **(B)** intermediate capillary plexus(ICP), and **(C)** deep capillary plexus(DCP). The changes of mean vessel density(%) at 0–1mm(central fovea), 1–3mm(inner ring), and 3–6mm(outer ring) are shown and compared between pre-infection and post-infection follow-ups. (*p <0.05, **p <0.01, ***p <0.001).

SS-OCTA analysis showed no significant changes in FAZ areas between baseline(mean[SD],0.339[0.097]) and the first(mean[SD],0.342[0.093],P=0.09) and second follow-up visits(mean[SD],0.344[0.098],P=0.052). However, mean(SD) inner retinal MVD(%) significantly increased from 60.76(2.88) at baseline to 61.59(3.72)(p=0.015) at the first follow-up and then decreased to the baseline level of 60.23(3.33)(p=0.16) at the second follow-up (**Figure 1**).

In the SCP, there was a significant rise in the mean(SD) MVD to 35.54(3.60) at the first follow-up from the baseline measurement of 34.90(3.31)(p=0.005). However, at the second follow-up, the mean(SD) MVD slightly decreased to 34.80(3.85), a change that was not statistically significant(p=0.685). Further analysis of the first follow-up data revealed that the increase in MVD was primarily observed in the outer ring(3–6mm) region, with mean(SD) MVD of 34.98(3.83) compared to the baseline value of 34.23(3.61)(p=0.001), no significant difference was found in the central fovea(mean[SD],9.09[3.18], p=0.100) and the inner ring(1–3 mm) region(mean[SD],40.67[4.39], p=0.372).

In the ICP, the mean(SD) MVD increased significantly to 24.14(3.23)(p=0.033) at the first follow-up and tended to decrease without a significance to 23.28(3.09)(p=0.536) at the second follow-up, compared to the baseline value of 23.51(3.22). Subgroup analysis of the first follow-up data revealed a significant increase in ICP MVD in both the central fovea (mean[SD],14.79[4.81] to 16.02[4.68], p=0.008) and the inner ring region (mean[SD],28.78[4.09] to 29.78[4.24], p=0.026), whereas no significant difference was detected in the outer ring region(mean[SD],22.26[3.59] to 22.76[3.64], p=0.104).

**Figure 2:**
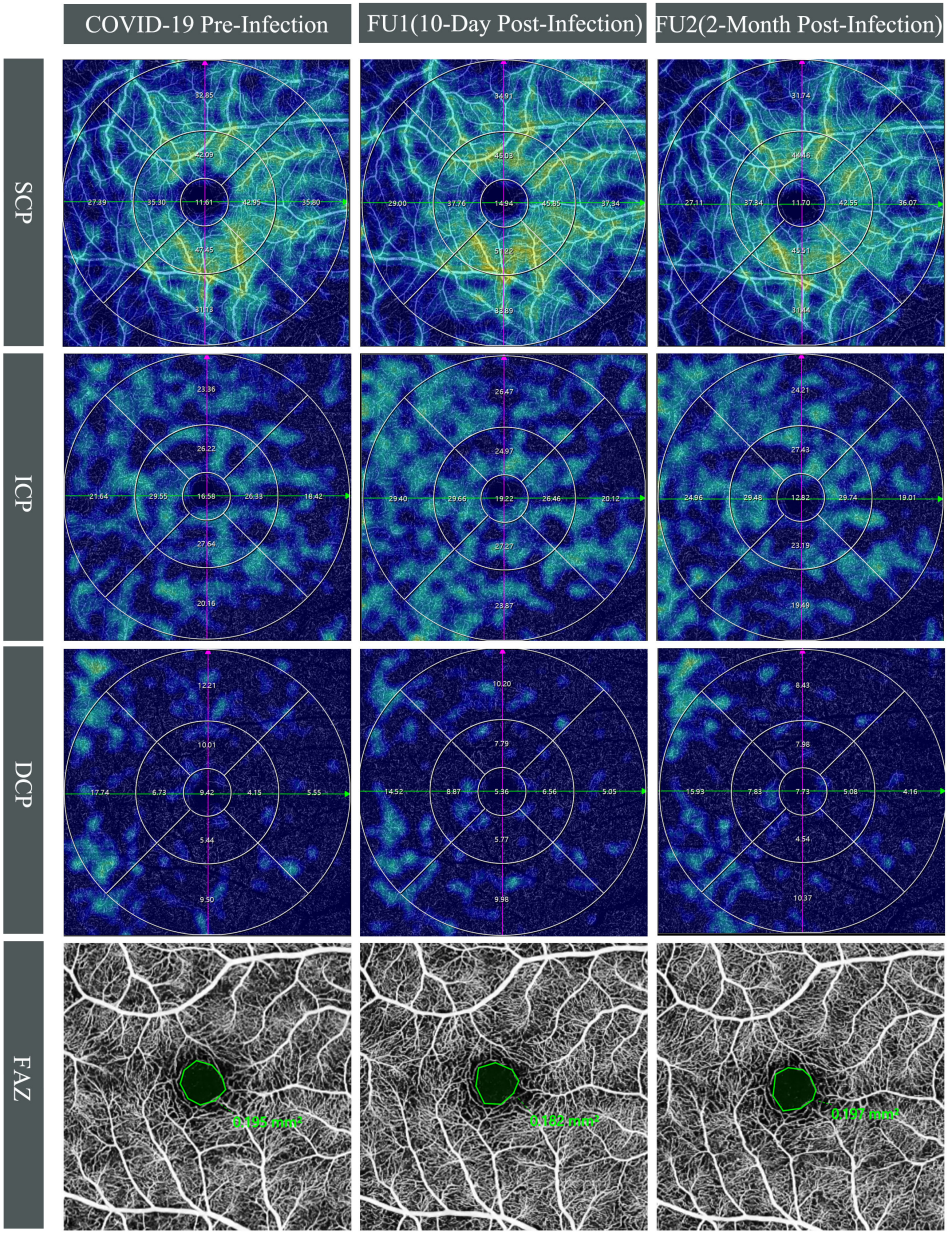
Longitudinal swept-source optical coherence tomography angiography(SS-OCTA) vessel density(VD) changes in the right eye of a 28-year-old male. Top three rows display SS-OCTA VD heatmaps of the superficial (SCP), intermediate (ICP), and deep capillary plexuses (DCP) across three time points: COVID-19 pre-infection, first follow-up (10-day post-infection), and second follow-up (2-month post-infection), which are overlaid by Early Treatment Diabetic Retinopathy Study grid. Compared to pre-infection, an increase in VD was observed at first follow-up for SCP (from 33.49% to 35.75%) and ICP (from 22.23% to 25.28%), whereas VD approached pre-infection levels with SCP at 33.46% and ICP at 22.89% at the second follow-up. The DCP exhibited a marginal decline at the first follow-up (from 10.16% to 9.21%) and further decreased at the second follow-up (8.92%). Bottom row shows the foveal avascular zone (FAZ) at COVID-19 pre-infection, 10-day post-infection, and 2-month post-infection. The FAZ measurements remained relatively unchanged pre- and post-COVID-19 infection, with values of 0.195 mm² at baseline, 0.182 mm² at the first follow-up, and 0.197 mm² at the second follow-up.

Regarding the DCP, a non-significant increase to 10.53(3.10)(p=0.285) was shown in MVD at the first follow-up, while a significant reduction of mean(SD) MVD to 9.84(3.14)(p=0.043) at the second follow-up compared to baseline value of 10.29(3.21). Although there was no significant difference in DCP at the first follow-up, subgroup analysis showed a significant increase in the inner ring region (mean[SD], from 7.11[3.16] to 8.08[3.10], p=0.002) (**Figure 2**).

For structural OCT parameters, a significant decrease in CMT, CV, and GCIPL was observed during the first visit(mean[SD], 250.67[16.83], 10.49[0.57], 77.36[6.43], respectively) compared to the baseline(mean[SD], 251.85[17.13], p<0.001; 10.51[0.57], p=0.039; and 77.79[6.42], p=0.002, respectively). These parameters recovered to pre-infection baseline level at the second follow-up(mean[SD], 251.60[17.08], p=0.401; 10.50[0.56], p=0.368; and 77.92[6.61], p=0.438, respectively). The detailed results of OCTA and OCT are shown in **Table 2**.

**Table 2:** Comparison of structural swept-source optical coherence tomography (SS-OCT) and swept-source optical coherence tomography angiography (SS-OCTA) parameters in COVID-19 patients before and after infection.

	COVID-19 Pre-Infection	FU1 (10-Day Post-Infection)		FU2 (2-Month Post-Infection)	
	mean[SD]	mean[SD]	p value#	mean[SD]	p value+
SS-OCT findings					
CMT(μm)	251.85[17.13]	250.67[16.83]	<0.001*	251.60[17.08]	0.401
Cube Volume	10.51[0.57]	10.49[0.57]	0.039*	10.50[0.56]	0.368
GCIPL(mm)	77.79[6.42]	77.36[6.43]	0.002*	77.92[6.61]	0.438
SS-OCTA findings					
FAZ area(mm2)	0.339[0.097]	0.342[0.093]	0.09	0.344[0.098]	0.052
VD(%)	60.76[2.88]	61.59[3.72]	0.015*	60.23[3.33]	0.162
SCP(%)	34.90[3.31]	35.54[3.60]	0.005*	34.80[3.85]	0.685
0–1mm	8.78[3.31]	9.09[3.18]	0.100	8.86[3.24]	0.745
1–3mm	40.36[4.14]	40.67[4.39]	0.372	40.14[4.39]	0.580
3–6mm	34.23[3.61]	34.98[3.83]	0.001*	34.16[4.11]	0.762
ICP(%)	23.51[3.22]	24.14[3.23]	0.033*	23.28[3.09]	0.536
0–1mm	14.79[4.81]	16.02[4.68]	0.008*	14.59[5.55]	0.685
1–3mm	28.78[4.09]	29.78[4.24]	0.026*	28.38[4.56]	0.473
3–6mm	22.26[3.59]	22.76[3.64]	0.104	22.08[3.40]	0.631
DCP(%)	10.29[3.21]	10.53[3.10]	0.285	9.84[3.14]	0.043*
0–1mm	2.86[2.81]	3.14[2.66]	0.192	2.64[2.81]	0.254
1–3mm	7.11[3.16]	8.08[3.10]	0.002*	6.71[3.03]	0.196
3–6mm	11.52[3.66]	11.54[3.59]	0.947	11.05[3.62]	0.070

*p < 0.05 as statistically significant and bolded.

#p values correspond to comparisons between COVID-19 pre-infection and 10-day post-infection.

+p values correspond to comparisons between COVID-19 pre-infection and 2-month post Infection.

CMT, Central Macular Thickness; COVID-19, coronavirus disease19; DCP, Deep capillary plexus; FAZ, fovea avascular zone; FU1, First Follow-up; FU2, Second Follow-up; GCIPL, Ganglion cells - inner plexiform layer; ICP, intermediate capillary plexus; SCP, superficial capillary plexus; SD, standard deviation.

## 4 Discussion

The study found no signs of clinical retinal microvasculopathy(retinal hemorrhage, CWS and tortuous vein) in participants who recovered from mild COVID-19 infections. However, SS-OCT/SS-OCTA demonstrated significant subclinical retinal microvascular alterations at 10 days post-COVID-19 infection with MVD increase and CMT, CV, and GCIPL decrease in macula region, while these alterations returned to the pre-infection level at 2-month post-infection. FAZ remained stable throughout the follow-up period. These findings suggested that COVID-19's impact on retinal microvasculature could be temporary and reversible in mild cases.

The impact of SARS-CoV-2 on retinal microvasculature still stands to be controversial. Some studies showed significant reduction in VD, increase in FAZ and CMT (19, 20), and thinning in GCIPL (14, 21) compared to healthy controls, indicating potential long-term consequences of the disease (22). Conversely, other studies found no notable differences in VD or mean FAZ measurements, similar to our results observed at the second follow-up (15, 23). The potential imbalance of preexisting conditions between COVID-19 patients and healthy controls, such as hypertension, diabetes and the compromised reliability of self-reported non-infected status in healthy controls was considered important factors in interpreting the discrepancy among previous studies (17). Our study, with a self-controlled design and laboratory-supported non-infected baseline status, minimized the effects of these confounding factors, more reflecting the impact of COVID-19 infection on retinal microvasculature.

Furthermore, this study revealed longitudinal changes of retinal microvasculature post-COVID-19 infection, with a transient increase of retinal blood flow in the early recovery stage that returned to baseline level two months later, indicating different results could be observed at different stages of the infection. Previous studies varied widely in the timing of ophthalmological examinations, with some reporting as early as 7–30 days after inpatient recovery and discharge (12, 19), and as late as 88 days after diagnosis (13), which may also be a reason for the inconsistent retinal microvascular findings.

Our 2-month post-infection follow-up results were aligned with some longitudinal studies, notably resembling the findings of Abrishami et al. (15). Their research compared macular microvasculature and FAZ parameters at two weeks, one month, and three months post-COVID-19 symptom recovery. They observed a reduction in mean VD for both SCP and DCP at one and three months compared to the initial post-recovery visit, with no significant changes in mean FAZ area. These trends were consistent with our 2-month post-infection findings compared with the early recovery phase. However, the increase in MVD observed during our 10-day post-infection follow-up has rarely been reported previously. This novel observation may be due to our pre-infection data collection, early follow-up timeline and the demographics of our study participants, who were young and had experienced mild infections. In previous longitudinal studies (11, 15, 21), the baseline acquisition was all performed post-infection, from COVID hospitalization to two weeks after discharge. Here, this study addresses the gap concerning early retinal vasculature changes due to SARS-CoV-2 infection. Furthermore, our first follow-up occurred at a mean of 10.9 days post-infection, making it the earliest follow-up recorded to our knowledge. At this point, acute effects of the SARS-CoV-2 virus, such as inflammation, could potentially persist even though PCR or antigen tests had already returned negative results, leading to an increase of MVD in the early recovery phase (24). The severity of the disease is another significant factor interpreting the results. Prior research has shown a considerable reduction in the central vascular area among moderate to severe COVID-19 patients compared to those with mild infections (16, 25). Given that our participants were young and mildly infected, it is less likely for retinal perfusion deficiencies to develop, keeping MVD remained at the pre-infection level 2 months after COVID-19 infection. Moreover, the Omicron variant, which was predominant during our research period, has lower pathogenicity (26, 27). Therefore, it may have a lesser impact on the retina compared to the original strain and Delta variant, which were prevalent in studies conducted in 2020 and 2021. Based on the fact that retinal microvascular changes were transient, we speculated our findings may reflect a physiological compensation of retinal blood flow in response to inflammation or hypoxia associated with COVID-19, and retinal perfusion deficits (increased FAZ or decreased VD) may occur when this autoregulatory mechanism fails.

Autoregulation of retinal microcirculation in response to systemic oxygen saturation has been reported in previous studies (28, 29). Hommer et al. (30) demonstrated that systemic hypoxia can lead to an increased VD in the SCP, while DCP remains stable. Beyond the systemic hypoxia caused by COVID-19 pneumonia, tissue hypoxia may be exacerbated due to the increased oxygen demand during inflammation and disrupted blood supply caused by thrombosis formed in hypercoagulable conditions induced by the SARS-CoV-2 virus. Inflammatory cells and fibrinous thrombi found in retinal and choroidal vessels of patients who died from COVID-19 suggested the occurrence of retinal hypoxia during the infection (31). Our findings are consistent with the observations of Hommer et al., showing a temporary surge in retinal blood flow, especially within the SCP and ICP. This suggests that the retina may adjust its blood flow in response to metabolic changes induced by COVID-19. Nonetheless, it remains unclear why increased VD was primarily noted in the inner ring of ETDRS grid in ICP and outer ring of ETDRS grid in SCP. The heightened vulnerability of the ganglion cell layer to hypoxia or ischemia could explain the more pronounced changes observed in the upper vascular layers (32). Moreover, the centripetally branching pattern of SCP, which supplies all other vascular plexuses vertically, may explain spatial differences in VD changes across different layers (32–34).

Another noteworthy finding in our study is the transient decrease of CMT, CV, and GCIPL at the 10-day follow-up, which recovered to pre-infection level at the 2-month follow-up. In previous studies, conflicting results were reported regarding CMT. While some groups reported an increased CMT (19, 20) in COVID-19 patients compared to controls, the majority found no significant difference (35–37). As to retinal volume, Kalaw et al. (22) found a significantly lower retinal volume of the outer third of the macula for severe COVID-19 patients. However, changes in the volume of the entire ETDRS grid for mild cases have not been reported, making them unavailable for comparison with our CV results (22). Temporal GCIPL thinning was also revealed by Seker et al (38) in patients shortly after recovering from COVID-19 and no difference in GCIPL thickness was obtained at a 12-month follow-up. Overall, our findings underscore the temporary impact of COVID-19 infection on retina, which does not appear to progress or persist over the long term. It is speculated that the consistent reduction of retinal thickness parameters in the early recovery phase of COVID-19 may be due to the mild asymptomatic systemic dehydration, which frequently accompanies COVID-19 (39). This dehydration may lead to neuroretina dehydration, allowing more fluid to drain into retinal vessels and thereby increasing blood flow during mild COVID-19 infection.

To our knowledge, this is the first longitudinal cohort study that closely tracks COVID-19 patients and compares retinal microvascular changes pre- and post-infection. Choosing medical staff as participants enabled us to capture retinal microvascular changes in the early recovery phase of COVID-19 infection for the first time. However, these alterations were found to return to pre-infection levels at the 2-month follow-up, which are more likely to represent the physiological adaptation of the retina in response to the SARS-CoV-2 virus. In this study, we conducted the final follow-up with the COVID-19 patients two months post-primary infection, considering that reinfection may occur three months post-infection, which could complicate the interpretation of the results. Indeed, COVID-19 reinfection is an often-overlooked factor in exploring the impact of COVID-19 on the retina. Although our results found no significant difference in MVD between 2-month post-infection and pre-infection, there was a tendency for MVD to decrease. It remains unclear whether retinal microvasculature can fully recover from every infection and whether multiple COVID-19 reinfections could have a cumulative effect on the retina. In longitudinal studies conducted by Bilbao-Malavé et al. (21) and Jevnikar et al. (11), long-term changes of retinal vasculature were aimed to evaluate at 6-month and one year respectively after hospital discharge. Both studies reported no significant changes in vessel density at these follow-up points compared to the initial post-infection baseline. However, neither study controlled for or identified potential confounding factors, such as COVID-19 reinfections or systemic comorbidities during the follow-up period. This oversight could dilute the accuracy of their results in representing the long-term consequences of COVID-19 on retinal microvasculature. Future research should be meticulously designed to specifically address the long-term impacts of COVID-19 reinfection on retinal microvasculature.

Some limitations of this study should be acknowledged. The generalizability of our findings might be limited due to the relatively small sample size, along with the participants' young age and mild infection. Older patients with severe COVID-19 infection and co-existing systemic comorbidities such as hypertension and diabetes were at a higher risk of retinal vascular complications. However, prospectively enrolling a large number of non-infected participants in a short time frame was challenging as China moved towards reopening, and predicting the severity of their potential infection was not feasible. Future research should focus on the impact of severe/hospitalized COVID-19 infections and COVID-19 reinfection on retinal microvasculature and the alterations in long COVID.

In conclusion, this study demonstrates the longitudinal changes in retinal microvasculature associated with COVID-19 infection. We observed no clinical retinal microvasculopathy features, such as retinal hemorrhage and CWS among patients recovering from mild COVID-19 infection. Transient alterations including increased retinal blood flow and reduced retinal thickness, were shown in the early recovery phase, which returned to baseline levels at the 2-month follow-up. This suggest that mild COVID-19 infection may have a temporary and reversible impact on retinal microvasculature.

## Data availability statement

The raw data supporting the conclusions of this article will be made available by the authors, without undue reservation.

## Ethics statement

This project was approved by the Institutional Review Board of Peking Union Medical College Hospital (study code K4168) and was conducted following the Declaration of Helsinki. Written informed consent was obtained from all participants.

## Author contributions

CZ: Conceptualization, Data curation, Formal analysis, Funding acquisition, Investigation, Methodology, Project administration, Software, Writing – original draft, Writing – review & editing. SC: Data curation, Formal analysis, Investigation, Methodology, Project administration, Software, Visualization, Writing – original draft, Writing – review & editing. HC: Writing – review & editing. JY: Writing – review & editing. YC: Conceptualization, Investigation, Project administration, Resources, Supervision, Writing – original draft, Writing – review & editing.
