# Supplementary figures and images for: New findings on retinal microvascular changes in patients with primary COVID-19 infection: a longitudinal study
Source: Front Immunol. 2024 May 21;15:1404785. doi: 10.3389/fimmu.2024.1404785 (PMC11148381; doi:10.3389/fimmu.2024.1404785)

**Supplementary**

**Figure1S** The flowchart of the study design.


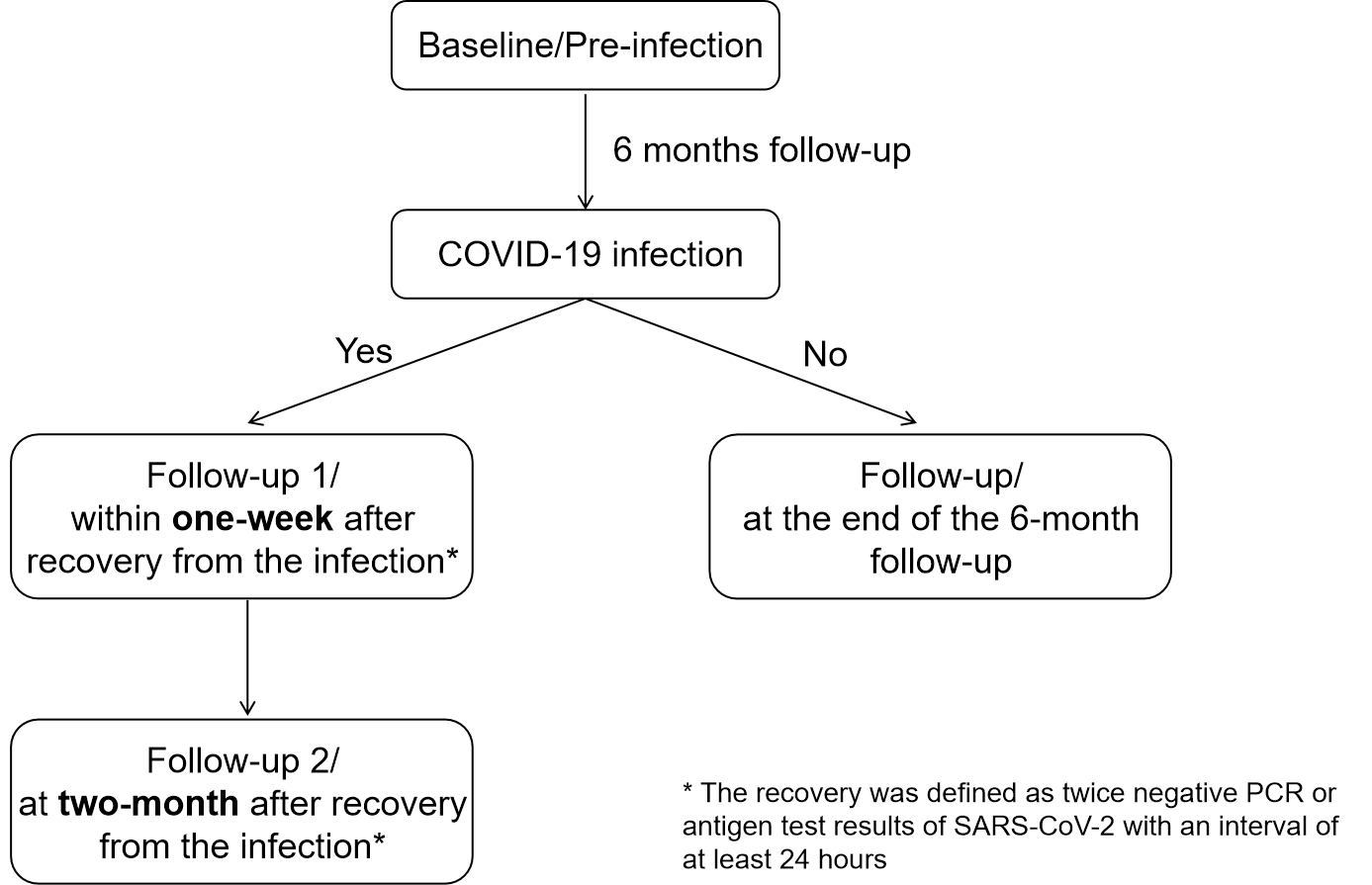

Supplement: Supplementary file 1 [file DataSheet_1.docx]
